# Gastro-Enterostomy and Pyloroplasty

**Published:** 1916-03

**Authors:** 


**Gastro-Enterostomy and Pyloroplasty.** J. M. T. Finney and Julius Friedenwald, Baltimore. 100 cases of each were studied. 67 of the former and 64 of the latter were operated on for obstruction, 44 and 51 respectively following gastric ulcer, 14 and 7 following duodenal ulcer, 9 and 6 following adhesions. There were 7 immediate deaths in the gastro-enterostomy cases, 5 in pyloroplasty cases. Immediately satisfactory results were noted in 82% of gastrostomies, 90% in pyloroplasties. Cases followed for two years reduced these percentages to 77.2 and 88.6 respectively. Secondary operations were required in 4 of each series. Hence they conclude that pyloroplasty is the operation to be favored, unless for special reasons, as inability to mobilize the duodenum on account of dense adhesions, thickening and infiltration about an ulcer. Excision of ulcers is often possible in pyloroplasty.

